# Drug affinity-responsive target stability unveils filamins as biological targets for artemetin, an anti-cancer flavonoid

**DOI:** 10.3389/fmolb.2022.964295

**Published:** 2022-08-25

**Authors:** Giusy Ferraro, Raffaella Belvedere, Antonello Petrella, Alessandra Tosco, Björn Stork, Stefano Salamone, Alberto Minassi, Federica Pollastro, Elva Morretta, Maria Chiara Monti

**Affiliations:** ^1^ Department of Pharmacy, Università di Salerno, Fisciano, Italy; ^2^ PhD Program in Drug Discovery and Development, Department of Pharmacy, Università di Salerno, Fisciano, Italy; ^3^ Institute of Molecular Medicine I, Medical Faculty and University Hospital Düsseldorf, Heinrich Heine University Düsseldorf, Düsseldorf, Germany; ^4^ Dipartimento di Scienze del Farmaco, Università del Piemonte Orientale, Novara, Italy; ^5^ PlantaChem Srls, Novara, Italy

**Keywords:** drug affinity-responsive target stability, proteomics, cytoskeleton, anti-cancer, bioactive natural compounds

## Abstract

Artemetin is a valuable 5-hydroxy-3,6,7,3′,4′-pentamethoxyflavone present in many different medicinal plants with very good oral bioavailability and drug-likeness values, owing to numerous bioactivities, such as anti-inflammatory and anti-cancer ones. Here, a multi-disciplinary plan has been settled and applied for identifying the artemetin target(s) to inspect its mechanism of action, based on drug affinity-responsive target stability and targeted limited proteolysis. Both approaches point to the disclosure of filamins A and B as direct artemetin targets in HeLa cell lysates, also giving detailed insights into the ligand/protein-binding sites. Interestingly, also 8-prenyl-artemetin, which is an artemetin more permeable semisynthetic analog, directly interacts with filamins A and B. Both compounds alter filamin conformation in living HeLa cells with an effect on cytoskeleton disassembly and on the disorganization of the F-actin filaments. Both the natural compound and its derivative are able to block cell migration, expectantly acting on tumor metastasis occurrence and development.

## 1 Introduction

**FIGURE 1:**
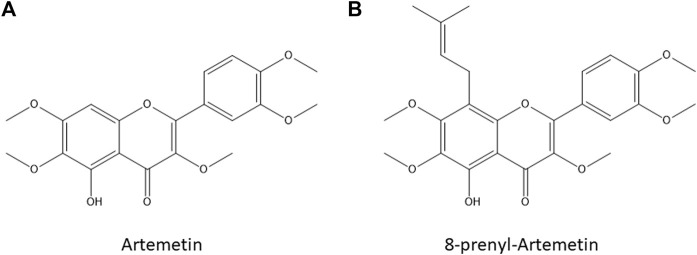
Artemetin [ART, Panel **(A)**] and 8-prenylartemetin [8-p-ART, Panel **(B)**] chemical structures.

Natural products (NPs) from plants and sponges have been studied since the earliest times, due to their therapeutic usefulness against various diseases, having weighty influences on pharmacological science. By tradition, nature has been an abundant source of diverse chemical entities that developed into novel lead molecules or therapeutics in contemporary drug discovery. Mostly, secondary metabolites of plants such as terpenoids, flavonoids, and alkaloids are well documented to hold anti-cancer properties (Choudhari et al., 2020). Indeed, they may positively or negatively control metabolic pathways altered in cancer cells such as proliferation, migration, and apoptosis through a multitude of action mechanisms. In addition, few undesirable side effects are ascribed to NPs possibly because of their resemblance with compounds found in the human diet which are highly tolerated (Rayan et al., 2017). Therefore, an in-depth investigation of the interactome of NPs of interest is a key step in the drug and target discovery course. Recent advances in coupling *omics* technologies and biochemical strategies are lastly changing the main approach to unveil the molecular mechanism of action of small molecules (Meissner et al., 2022). Indeed, proteins represent the majority of targets of almost all drugs, and they are regulated at multiple levels, mainly by non-covalent or covalent interactions with other ligands varying in their activity and conformation. Over the years, mass spectrometry (MS)-based proteomics has advanced from classifying proteins in biological samples for evaluating their functional modulation by quantitatively measuring protein alteration related to drug treatment (Schenone et al., 2013). Specifically, chemo-functional proteomics empowers biochemical or biophysical procedures to detect direct interactions of a bioactive metabolite with its partners, mainly using thermal stability-based approaches or enlarged resistance to proteolysis (Lomenick et al., 2009; Feng et al., 2014). Indeed, NPs binding to targets affect protein conformation and alter their susceptibility to proteolytic action by stabilization of the folded state: drug affinity-responsive target stability (DARTS) monitors the reduction in protease exposure of the target protein(s) upon direct binding of the molecule of interest. Similarly, limited proteolysis-coupled mass spectrometry (LiP-MS) measures proteolytic peptides that play as reporters to determine which protein regions are hugely affected by the NP interaction (Morretta et al., 2017; Schopper et al., 2017; Del Gaudio et al., 2018; Hwang et al., 2020). In this work, we applied our consolidated DARTS and LIP-MS platform (Ceccacci et al., 2020; Morretta et al., 2021a) to disclose the interactome of an attractive bioactive NP such as artemetin (ART), a 5-hydroxy-3,6,7,3′,4′-pentamethoxyflavone (Figure 1A) found in many different medicinal plants such as *Achillea millefolium L.*, *Artemisia absinthium*, *Artemisia gorgonum*, *Cordia verbenacea*, *Vitex trifolia*, and *Vitex negundo* (Ortet et al., 2008; De Souza et al., 2011; De Almeida et al., 2016; Wee et al., 2020; Martim et al., 2021; Sichaem et al., 2021).

ART has excellent oral bioavailability and drug-likeness values (Lee et al., 2018), and it possesses numerous bioactivities, such as anti-inflammatory (Sertie et al., 1990; Sertié et al., 1991; Grossini et al., 2015; Wee et al., 2020), anti-microbial, and anti-malarial even if showing milder anti-plasmodial power than artemisin (Liu et al., 1992; Ortet et al., 2011; Tasdemir et al., 2015), hypotensive (De Souza et al., 2011), and anti-cancer ones (Li et al., 2005; Kim et al., 2013; Martins et al., 2014; Sinha et al., 2015; Rosa et al., 2022). ART anti-inflammatory activity has been studied both at a cellular level and *in vivo*. Mainly, ART enlarged eNOS-dependent NO production by mechanisms linking muscarinic receptors, *β*2-adrenoreceptors, and kinases and upgraded cell viability in porcine aortic endothelial cells by counteracting apoptosis and through the modulation of mitochondria, acting as an antioxidant agent (Grossini et al., 2015). ART significantly suppressed TNF-α and IL-1β production (Wee et al., 2020) and inhibited carrageenan-induced paw edema (Sertie et al., 1990). Vice versa, the anti-cancer activity of this molecule has been disclosed in less detail: ART has been found to have a good anti-proliferative effect on many cancer cells, inducing apoptosis of human myeloid leukemia K562 cells and tsFT210 cells through weak inhibition of the cell cycle at the G2/M phase (Li et al., 2005). Moreover, it has been found that ART showed significant cytotoxic activity against human breast cancer cells (MCF-7 and T47D), modulating microtubule depolymerization by activating the mitotic spindle checkpoint and leading to cell apoptosis.

However, ART targets in cancer cells have not been truly recognized, and thus we moved to investigate its complete interactome in HeLa cell lysates. It has been recently reported that eupatilin, a chemical analog of ART, showed a cytotoxic effect on cancer HeLa cells (Rosa et al., 2020) and the effect of ART and 8-prenylated-artemetin (8-p-ART, Figure 1B) has been investigated on this cell line (Salamone et al., 2021). As reported by Salamone et al. (2021), ART and 8-p-ART were able to affect HeLa cell viability at concentrations higher than 25 µM after 72 h of incubation. Our preliminary results reported in Supplementary Figure S1 are fully in line with those reported in Salamone et al. (2021). Moreover, both compounds changed the cell lipid composition, thus affecting the lipid metabolism which is recognized as a novel anti-cancer target. Moreover, phase-contrast images of HeLa cells after 72-h treatment with both molecules showed some changes in the cell morphology compared to control cells, mainly in terms of cell elongation (Salamone et al., 2021). Thus, since it was demonstrated that the prenylation hardly enlarged the cytotoxic effect in cancer HeLa cells, and also increasing the alteration of cell phospholipid and fatty acid composition, the 8-p-ART isomer has also been tested here in comparison with ART in cell assays.

## 2 Materials and methods

ART and 8-p-ART were a kind gift from PlantaChem Srls. A purity of 98% has been reported based on HPLC analysis with RP C-18, as reported by Salamone et al. (2021).

### 2.2 Identification of artemetin cellular targets

#### 2.2.1 Cell culture

HeLa cells (ATCC^®^ CCL-2™; Manassas, VA United States) were grown in Dulbecco’s modified Eagle medium (DMEM) with 10% fetal bovine serum (FBS) and 1% penicillin and streptomycin (Euroclone, Milan, Italy). The cells were grown at 37°C in humidified air at 5% CO_2_.

#### 2.2.2 Drug affinity-responsive target stability

HeLa cells were lysed in PBS supplemented with 0.1% v/v Igepal and with a protease inhibitors mixture (final concentration 1x, Sigma-Aldrich). The lysate was centrifuged at 10,000 g for 10 min (4°C), and the protein concentration of the obtained supernatant was determined by Bradford assay. Then, DARTS experiments were carried out: different concentrations of ART or 8-p-ART (1 μM, 10, and 100 μM) were incubated with 300 μg of HeLa cell lysate for 1 h at room temperature under rotary shaking. The samples were then submitted to limited proteolysis, for 30 min at 25°C, at a ratio of 1:1500 w/w of subtilisin (Sigma-Aldrich) with respect to protein amount. Two aliquots of cell lysates were treated with DMSO and one of them with subtilisin, as control. Then, the protease was blocked by adding PMSF (phenylmethylsulfonyl fluoride, Sigma-Aldrich, 1 mM final concentration) to each sample. Then, all aliquots were boiled in Laemmli buffer (60 mM Tris–HCl pH 6.8, 2% SDS, 0.001% bromophenol blue, 1% glycerol, and 2% *ß*-mercaptoethanol), and 20 μg were loaded on a 4-12% Bis–Tris Criterion™ XT Precast Gel (Bio-Rad Laboratories S.r.l.), which was then stained by Coomassie solution and submitted to a densitometric analysis through ImageJ. These experiments were carried out in triplicate for ART at different concentrations, and incubating with 8-p-ART at the same ART concentrations. Protein bands were cut out from the gels and submitted to an *in situ* tryptic digestion protocol (Shevchenko et al., 2006). Briefly, gel slices were reduced with DTT (1,4-dithiothreitol), alkylated with IAA (iodoacetamide), washed, and rehydrated on ice for 1 h in 12 ng/μl trypsin solution. Then, the enzyme excess was removed and replaced with ammonium bicarbonate (Ambic, 50 mM; pH 8.5), allowing protein digestion to proceed overnight at 37°C. Subsequently, the supernatants were collected, and peptides were extracted from each gel slice, shrinking them in 100% ACN (acetonitrile). The obtained peptides mixtures were dried under vacuum and dissolved in formic acid (FA, 10%) for the LC-MS-MS analysis.

#### 2.2.3 Nano-LC-MS-MS analysis

A volume of 1 µl of each peptide sample was injected into a nano-UPLC system (Thermo Fisher Scientific, Bremen) separating peptides on an EASY-Spray PepMAP™ RSLC C18 column (3 µm, 100Å, 75 μm × 50 cm, Thermo Fisher Scientific, Bremen) at a flow rate of 0.3 ml/min. Peptide elution was achieved at a flow rate of 300 nL/min with the following gradient: 1 min at 3% B, 1–40 min to 28% B, 40–41 min to 70% B, 41–49 min at 70% B, and 50–60 min back to 3% B (A: 95% H_2_O, 5% ACN, and 0.1% AcOH; B: 95% ACN, 5% H_2_O, and 0.1% AcOH). The mass spectrometer was operated in the data-dependent acquisition mode (DDA). Full scan MS spectra were acquired with the following settings: scan range 400–2000 m/z, full-scan automatic gain control (AGC) target 1e6 at 70,000 resolution, and maximum injection time 100 ms. MS/MS spectra were generated for up to 10 precursors (normalized collision energy of 30%), and the fragment ions were acquired at a resolution of 17,500 with an AGC target of 1e5 and a maximum injection time of 50 ms.

#### 2.2.4 Bioinformatics analysis of targets

Subsequently, database searches were carried out on Mascot Deamon (Mascot Server Version 2.5), employing the SwissProt database and the following settings: two missed cleavages; carbamidomethyl (C) as fixed modification; oxidation (M) and phosphorylation (ST) as variable modifications; peptide tolerance 30 ppm; and MS/MS tolerance 0.8 Da (see also https://www.matrixscience.com/daemon.html).

### 2.3 Immunoblotting analysis

The DARTS samples of ART were submitted to Western blotting analysis. First, 20 µg of each sample were loaded on an 8% SDS-PAGE and transferred onto a nitrocellulose membrane; then, they were incubated for 1 h in a blocking solution (5% w/v milk in TBS-t: 31 mM Tris pH 8, 170 mM NaCl, 3.35 mM KCl, and 0.05% Tween 20) and left for 16 h at 4°C with monoclonal antibodies against FLNA and FLNB (1:1000 v/v, RayBiotech). Then, a rabbit peroxidase-conjugated secondary antibody (1:1000 v/v, Thermo Fisher Scientific) was added, and the signal was detected using an enhanced chemiluminescent substrate and LAS 4000 digital imaging system. Finally, an antibody against glyceraldehyde 3-phosphate dehydrogenase (GAPDH, 1:2000 v/v, Invitrogen) in 5% milk was used as a loading normalizer.

### 2.4 Filamins A and B LiP-MRM method building

Filamin A (FLNA) and filamin B (FLNB) tryptic peptides were selected through the proteomics data resource *PeptideAtlas* on its human build and queried into the *complete Human SRM Atlas* (https://db.systemsbiology.net/sbeams/cgi/PeptideAtlas/GetTransitions) in order to identify the best daughter ions for each parent ion. Thus, complete methods listing filamin peptides and their three best transitions were listed, and a HeLa lysate tryptic digest was run using these methods. Before that, the HeLa cell lysate was submitted to an in-solution digestion protocol: proteins were denatured with 8 M urea/50 mM Ambic (4 M final urea concentration), and disulfide bonds were reduced with 10 mM DTT for 1 h at 25°C and 800 rpm and then alkylated with 20 mM iodoacetamide for 30 min at 25°C and 800 rpm, in the dark. Iodoacetamide was quenched with 10 mM DTT (10 min, 25°C, and 800 rpm), and urea was diluted to 1 M with 50 mM Ambic before adding trypsin/LysC solution (Promega, Madison, Wisconsin) at the enzyme to proteins ratio of 1:100 w/w. Digestion proceeded overnight at 37°C under continuous shaking and was then quenched by adding FA to lower the pH to 3. The peptide mixture was then dried under vacuum, dissolved in 1 ml 5% FA, and desalted through a Sep-Pak C18 1 cc (50 mg) cartridge (Waters, Milford, MA, United States). The cartridge was activated by flushing with 3 ml of 100% ACN and then conditioned with 3 ml of 0.1% FA. The sample was then loaded, desalted by flushing the cartridge with 3 ml of 0.1% FA, and finally eluted by flushing two times with 500 μl of 80% ACN, 20% H_2_O, and 0.1% FA. For the subsequent MS analysis, the peptide mixture was dried under vacuum and re-dissolved in 10% FA. UPLC-ESI-MRM-MS analyses were performed on a 6500 Q-TRAP UPLC MS/MS system from AB Sciex equipped with Shimadzu LC-20A and auto sampler systems. UPLC separation was performed on a Kinetex PS C18 column (50 × 2.1 mm, 2.6 μm, 100 Å, Phenomenex, Torrance, United States), using 0.1% FA in H_2_O (A) and 0.1% FA in ACN (B) as mobile phases and a linear gradient from 5 to 95% of B over 20 min (flow rate: 300 μl/min). Q-TRAP 6500 was operated in the positive MRM scanning mode, with a declustering potential (DP) set at 80 V, entrance potential (EP) at 10 V, and cell exit potential (CXP) at 22 V. Collision energy (CE) was calculated for each precursor as follows: CE = 0.044 × (Q1 m/z) + 5.5 (precursor charge 2+) and CE = 0.051 × (Q1 m/z) + 0.5 (precursor charge >2+).

### 2.5 Artemetin/filamin interaction feature evaluation through t-LiP-MRM

HeLa cells lysate were incubated with DMSO or ART (10 and 100 μM final concentrations), for 1 h at room temperature. The samples were then submitted to limited proteolysis with 1:500 and 1:1500 (w/w) ratios of subtilisin with respect to the protein amount, leaving a DMSO-treated aliquot undigested to be kept as a positive control. Subtilisin was then quenched with PMSF (1 mM final concentration), and the mixtures were shifted to denaturing conditions adding urea (4 M final concentration) to perform *in-solution* digestion and desalting, as previously reported (Morretta et al., 2021a). The samples were then injected into the previously reported UPLC-ESI-MS system and analyzed through the optimized filamins MRM methods. Each protein tryptic peptide peak area was then measured using Analyst Software from AB Sciex. Each sample was analyzed in duplicate.

### 2.6 Artemetin and 8-prenyl-artemetin PAMPA assays

Donor solution (50 μM) was prepared by diluting 5 mM ART or 8-p-ART stock solutions using phosphate saline buffer (PBS: 137 mM NaCl, 2.7 mM KCl, 10 mM Na_2_HPO_4_, and 2 mM KH_2_PO_4_; pH 7.4). The filter membrane was coated with 5 μL of specific lipid solution prepared as 1% w/v phosphatidylcholine solution in n-dodecane. Donor solution (150 μl) was added to each well of the filter plate. To each well of the acceptor plate, 300 μl of solution (5% DMSO in PBS) was added. The donor and acceptor plates were assembled to obtain a sandwich and incubated for 24 h at room temperature under gentle shaking. Then, the sandwiched plates were separated, and 250 μl of the solution from the acceptor plate and 100 μl of the solution from the donor one were transferred to a new multi-well plate, and the absorbance was measured by UV spectroscopy using a Multiscan GO microplate spectrophotometer (Thermo Scientific) at 250–500 nm (5 nm steps). The permeability value Log Pe was determined. The membranes’ integrity was checked using propranolol and furosemide as control molecules.

### 2.7 Confocal microscopy

HeLa cells were seeded in a glass-bottomed multi-well plate at 8.0 × 10^4^. After treatment with either ART or 8-p-ART, cells were fixed in p-formaldehyde at 4% v/v in PBS (Lonza; Basilea, Swiss), permeabilized with Triton X-100 at 0.5% v/v in PBS (Lonza; Basilea, Swiss), and then blocked with goat serum at 20% v/v in PBS (Lonza; Basilea, Swiss). Incubation with the respective antibodies against FLNA and FLNB was performed O/N at 4°C. F-actin detection was evaluated by phalloidin-FITC (5 μg/ml, Sigma-Aldrich; Saint Louis, MO, United States) for 30 min at RT in the dark. The staining with conjugated secondary antibodies (1:500, anti-mouse), the nuclei with DAPI (1:1000), and the subsequent confocal microscope analysis were performed as described in Belvedere et al. (2018a) and Belvedere et al. (2018b).

### 2.8 Wound-healing assay

The confluent monolayer of HeLa was scraped using a pipette tip to produce a wound. Next, cells were treated for 24 h with 25 µM ART or 8-p-ART following a previous administration of mitomycin C (10 μg/ml, Sigma-Aldrich; Saint Louis, MO, United States) to ensure the block of mitosis. The wounds were photographed and analyzed as previously reported (Belvedere et al., 2017; Franco et al., 2020). Briefly, the wounded cells were incubated at 37°C in a humidified and equilibrated (5% v/v CO_2_) incubation chamber of an Integrated Live Cell Workstation Leica AF-6000 LX. A ×10 phase contrast objective was used to record cell movements with a frequency of acquisition of 10 min for each experimental condition. The migration rate of individual cells was determined by measuring the wound closure from the initial time (0 h) to the selected time points (over 24 h) (bar of distance tool, ASF software, Leica, Wetzlar, Germany). For each wound, five different positions were registered, and for each position, ten different cells were randomly selected. Carrying on with the images taken every 10 min, the made distance was recorded each 2 h.

### 2.9 Statistical analysis

All the biological assay data and statistical analyses were made with Microsoft Excel. We report the number of independent repetitions and *p*-values in the legends of the figures for each experiment. All results are the mean ± standard deviation of at least three experiments performed in triplicate. The statistical data analysis was performed using the two-tailed *t*-test for comparing two variables, and the differences were considered significant if *p* < 0.05, *p* < 0.01, and *p* < 0.001.

## 3 Results

### 3.1 Identification of artemetin cellular targets through DARTS

To identify ART cellular-interacting proteins, DARTS experiments were performed. This approach is based on limited proteolysis of a cellular lysate, pre-treated or non-treated with the compound of interest, by a low-specificity protease as subtilisin, under native conditions. SDS-PAGE of the samples permits inspecting which proteins better resisted the enzymatic cleavage: the band intensity of the putative protein target(s) rises in presence of the small molecule, due to its protective effect, in a concentration-dependent fashion. Thus, the target protein(s) can be identified through *in situ* digestion, nano-UPLC-MS/MS, and bioinformatics tools.

**FIGURE 2:**
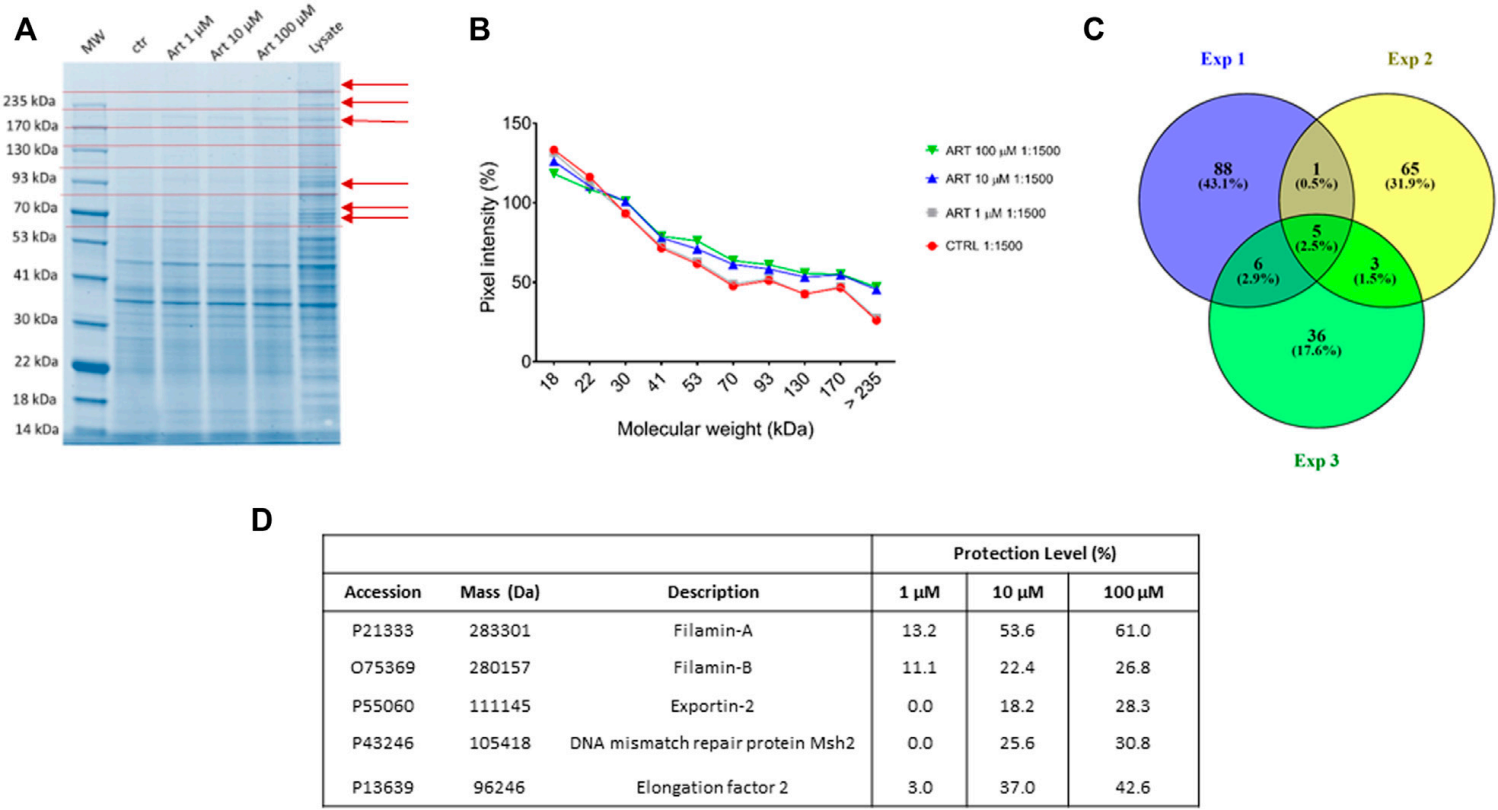
**(A)** Coomassie-stained gel showing protection of proteins (red arrows) to protease upon ART interaction. Red dotted lines indicate gel cutting patterns. **(B)** SDS-PAGE densitometric analysis. **(C)** Venn diagram showing shared ART-protected proteins among three DARTS experiments. **(D)** Mascot-retrieved protection percentages of the five proteins shared among all the DARTS experiments.

Here, native HeLa cell samples were mixed with increasing ART amounts and then subjected to subtilisin-assisted limited proteolysis. One sample was treated with the vehicle and represented the negative control. Another one was kept undigested and without ART as a positive control. All samples were submitted to SDS-PAGE followed by Coomassie blue staining, and all gel lanes were digested, principally those bands whose intensity raised at increasing ART concentrations (Figure 2 Panels A and B). The nano-UPLC-MS/MS analysis of the digested samples provided protein identification using the Mascot database search. All the experiments were carried out in triplicate: proteins identified in all DARTS were considered (Figure 2 Panel C) to identify ART-interacting ones by comparing the Mascot score outputs with both the positive and negative control samples. Indeed, Mascot scores are related to the protein identification confidence: higher scores reflect a better match of the experimental spectra to *in silico* MS and MS/MS ones due to a higher abundance of the intact protein in the gel lane. Thus, ART protection levels (reported as percentages) were evaluated for each identified protein. Among them, filamins A (FLNA) and B (FLNB) were selected as the main partners since they were better protected from proteolysis in all DARTS replicates (Figure 2 Panel D and, for more details, Supplementary Table S1).

**FIGURE 3:**
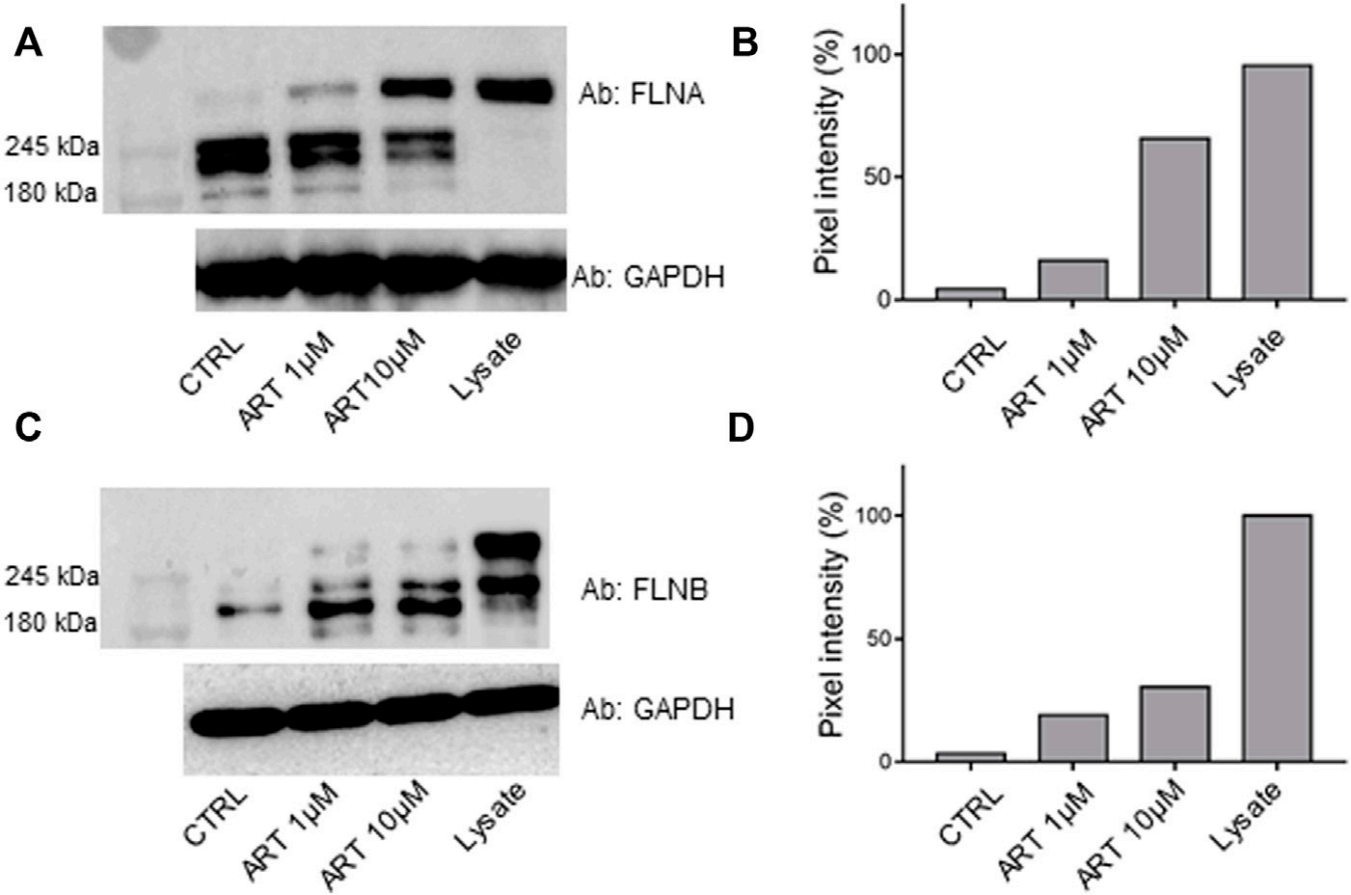
lmmunoblotting analysis of one of the DARTS experiments revealing FLNA [panel **(A)]** and FLNB [panel **(C)**], together with their densitometric analysis [panels **(B,D)**, respectively]. GAPDH is resistant to subtilisin under these experimental conditions and is used as a loading control.

Since both FLNA and FLNB were the most protected targets at the low ART concentration, a bio-orthogonal validation was planned to use a more quantitative technique such as immunoblotting. Thus, the direct interaction between these two putative partners and the small molecule was unequivocally confirmed by Western blotting analysis, submitting all DARTS samples to an anti-FLNA and FLNB antibody reaction (Figure 3). Actually, comparing immunoblotting signals corresponding to undigested FLNA and FLNB, it is clear that the intact proteins’ signals increase their intensity according to ART concentration (MW∼280 kDa). An opposite trend could be observed for the signal corresponding to the first FLNA proteolytic fragments (at ∼245 and ∼200 kDa), which decreases with ART concentration. In contrast, a protective effect on the first FLNB proteolytic fragments can be detected, suggesting the binding of ART to FLNA and FLNB in different ways. The ART binding to the other targets, namely, elongation factor 2 (EF2), exportin-2 (EXP2), and DNA mismatch repair protein (MSH2) has been detailed in Supplementary Figure S2, showing a less remarkable protein protection. Among these three proteins, EF2 seems the more affected by ART: this is a very promising potential target due to its implication in many cellular pathways. Indeed, the regulation of EF2 touches protein synthesis, protein translocation reactions in eukaryotes, and energy conservation under nutrient-deprived conditions which are critical for numerous biological phenomena, such as neurodegeneration and cancer (White-Gilbertson et al., 2009; Kaul et al., 2011).

### 3.2 Analysis of the interaction features of FLNA and FLNB binding to artemetin by the targeted limited proteolysis approach

**FIGURE 4:**
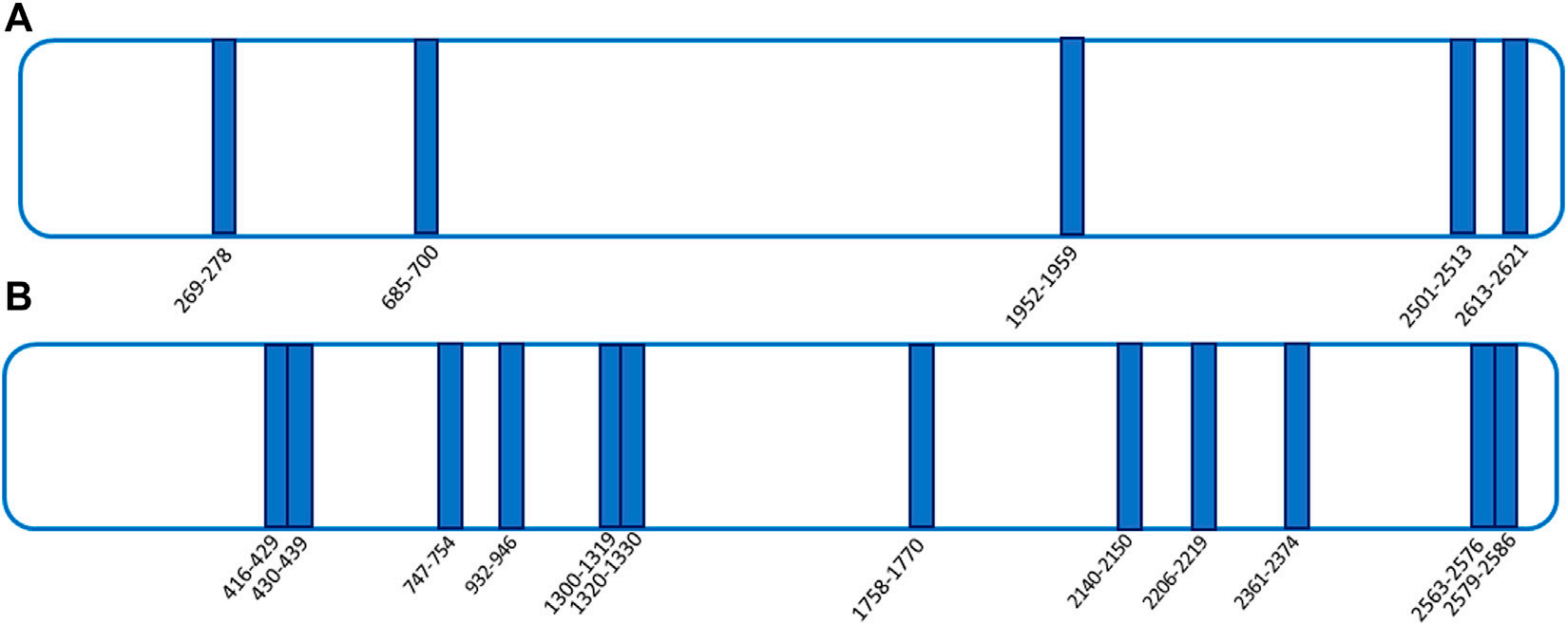
t-LiP-MRM experimental results. ART-protected peptides are reported as blue bars in both FLNA [panel **(A)]** and FLNB [panel **(B)]** schematic representations.

To get a deep understanding of the conformational changes induced by ART on its targets, a t-LiP multiple reaction monitoring (MRM) setup was performed. T-LiP MRM aims to identify which protein regions are involved in the target/ligand-binding event in a whole-cell lysate, looking at the protein alterations due to a bound ligand. The HeLa cells’ native proteins were treated or non-treated with ART, and then double-protease digestion was carried out: first, subtilisin-limited proteolysis was achieved under native conditions, and then full tryptic digestion was settled in denaturing settings. This action produced a mixture of semi-tryptic and fully tryptic peptides, the latter suitable for targeted MRM-MS quantification analysis. Indeed, the area of fully tryptic peptides is symptomatic of the local target structural changes due to ligand binding: the area of the single peptide is higher when subtilisin-limited proteolysis is less effective due to the ART interaction (Feng et al., 2014). An initial *in silico* search using the bio-informatics devices, *Peptide Atlas* and *SRM Atlas*, is performed to set the best MRM transitions of FLNA–FLNB theoretically fully tryptic peptides and to map the protein sequence. Initially, a cell lysate sample was denatured and extensively proteolyzed by trypsin to unequivocally recognize the peptide signals and their most intense daughter ions by LC-MRM-MS. Next, native protein mixtures were incubated with ART (10 and 100 μM) or vehicle and treated with subtilisin under restricted conditions of time, temperature, and at the enzyme to proteins ratios of 1:500 and/or 1:1500 (w/w). Following this, the samples were denatured and fully digested by trypsin, and the mixtures were run on the LC-MRM-MS system to quantify the area of each FLNA and FLNB tryptic peptide. Those peptides mapping for FLNA and FLNB were analyzed by comparing the controls and the treated samples: peptides with increased intensity in the samples exposed to the small molecule were considered diagnostic of ART protection on specific FLNA and FLNB regions (Supplementary Table S2). As shown in Figures 4A,B, ART interacts differently with FLNA and FLNB; in particular, the main FLNA-protected regions were the N-terminal and the C-terminal, which are the actin-binding domain and the self-association site tail interacting with FLNB, respectively. Differently, more peptides have been protected by ART along the entire sequence of FLNB: five of them fall in the C-terminal region, deputed to the interaction with FLNA. The C-terminal is also the region involved in the dimerization of filamins allowing the formation of a V-shaped flexible structure that is essential for their function (Feng and Walsh, 2004).

### 3.3 Artemetin and 8-prenyl-artemetin permeation of artificial membranes

Before moving to assays in live cells, we tested ART and its supposed more permeable 8-prenyl derivative in the parallel artificial membrane permeability assay (PAMPA) to measure their effective permeability (expressed as -Log Pe) through an artificial lipid membrane (Le Roux et al., 2020). Propanolol and furosemide at 250 μM were used as positive and negative control molecules giving a -Log Pe of 5.30 for propranolol and 6.90 for furosemide (−Log Pe < 6 is considered good permeability and −Log Pe > 6.5 as impermeable). In this assay, ART displayed a good propensity to cross the membrane *in vitro*, with a -Log Pe of 5.87 ± 0.01; as expected, 8-p-ART showed an even better permeability profile with a -Log Pe of 5.57 ± 0.01.

### 3.4 8-Prenyl-artemetin targets filamin A and filamin B

To prove that the prenylation on ART C8 did not alter the interaction with FLNA and FLNB, a DARTS-based experiment was performed using 8-p-ART under the same experimental conditions used for ART, as described previously, followed by nano-UPLC-MS-MS analysis and Mascot identification. As expected, 8-p-ART is able to well protect both FLNA and FLNB already at the lowest tested amount, even if without a concentration-dependent fashion (Table 1 and Supplementary Table S1 for more details). This is fully in line with the next results showing a higher effect of the 8-prenyl derivative.

**TABLE 1:** Mascot-retrieved protection percentages of filamins A and B in a DARTS experiment carried out on HeLa cell lysates incubated with 8-p-ART at the reported concentrations.

8-p-ART			Protection level (%)		
Accession	Mass (Da)	Description	1 μM	10 μM	100 μM (l)
P21333	283,301	Filamin-A	53.2	42.7	55
O75369	280,157	Filamin-B	53	39.3	47

### 3.5 Effects of artemetin and 8-prenylated artemetin on filamins A and B and F-actin in HeLa cells

**FIGURE 5:**
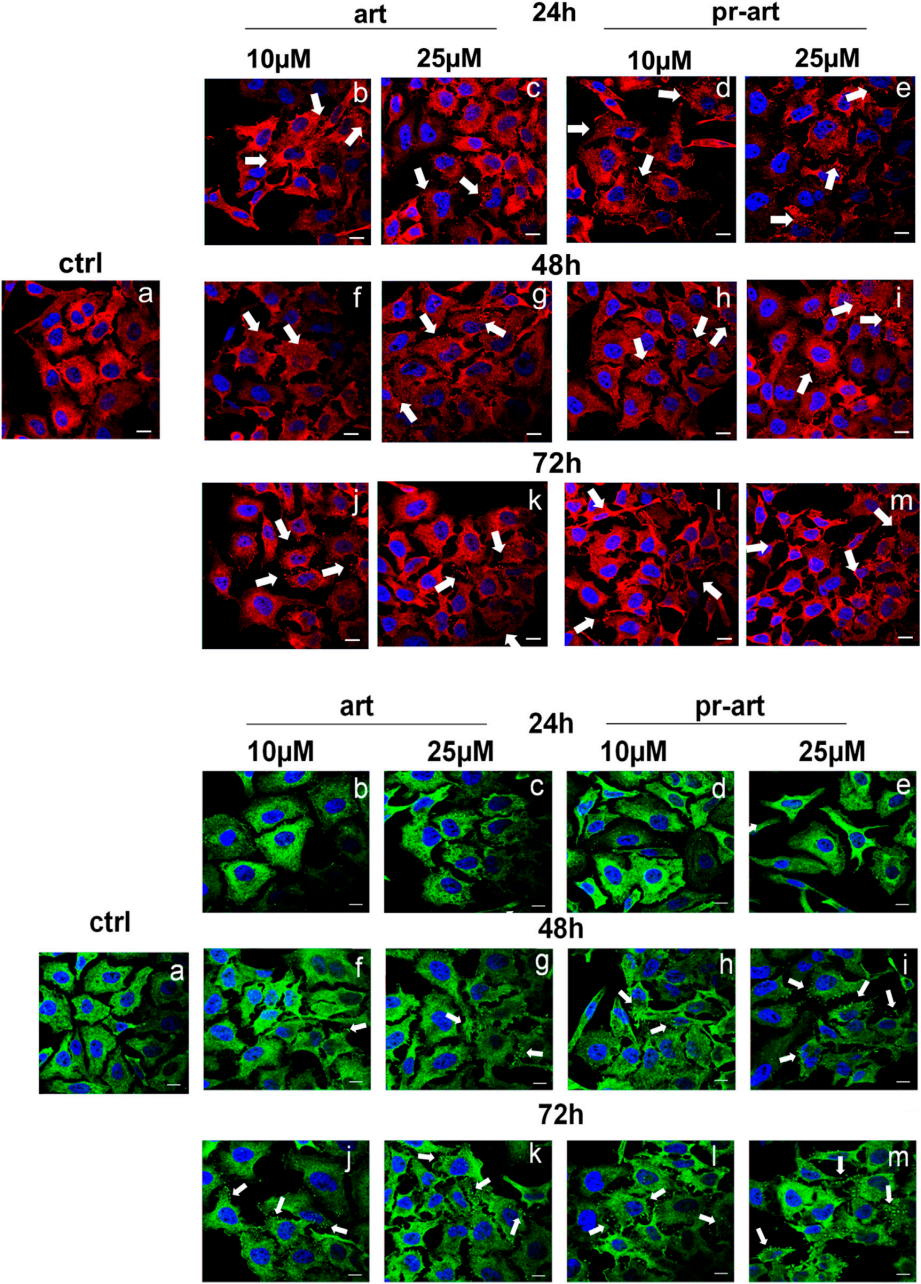
lmmunofluorescence analysis of ART and 8-p-ART-treated (10 and 25 μM; from 24 to 72 h) HeLa cells. These ones were fixed and labeled with an antibody against FLNA (red) and FLNB (green). White arrows show FLNA and FLNB disorganization. Magnification: ×63 1.4 NA. Bar = 100 µm. All images are representative fields of n = 3 experiments with similar results.

To monitor the interaction of ART and its prenylated form with FLNA and FLNB in living HeLa cells, confocal microscopy was performed. The immunofluorescence staining of FLNA showed that, in the presence of ART and 8-p-ART, this protein appeared disassembled in pointed structures starting from 24 h of treatments (Figure 5, upper panels b–e, white arrows). This effect was more evident in the case of 48 h of treatments with both substances (Figure 5, upper panels f–i, white arrows) to remain constant at 72 h (Figure 5, upper panels j–m, white arrows). There are no significant differences between ART and its relative prenylated form, and the highest concentration (25 µM) of both compounds showed the strongest activity. The same tendency was highlighted in FLNB (Figure 5, lower panels, white arrows). In particular, after 48 h from the addition of ART and 8-p-ART, this protein began to form fragmented structures, mainly at 25 µM of both molecules (Figure 5, lower panels f–m, white arrows), indicating a cytoskeletal disassembling.

**FIGURE 6:**
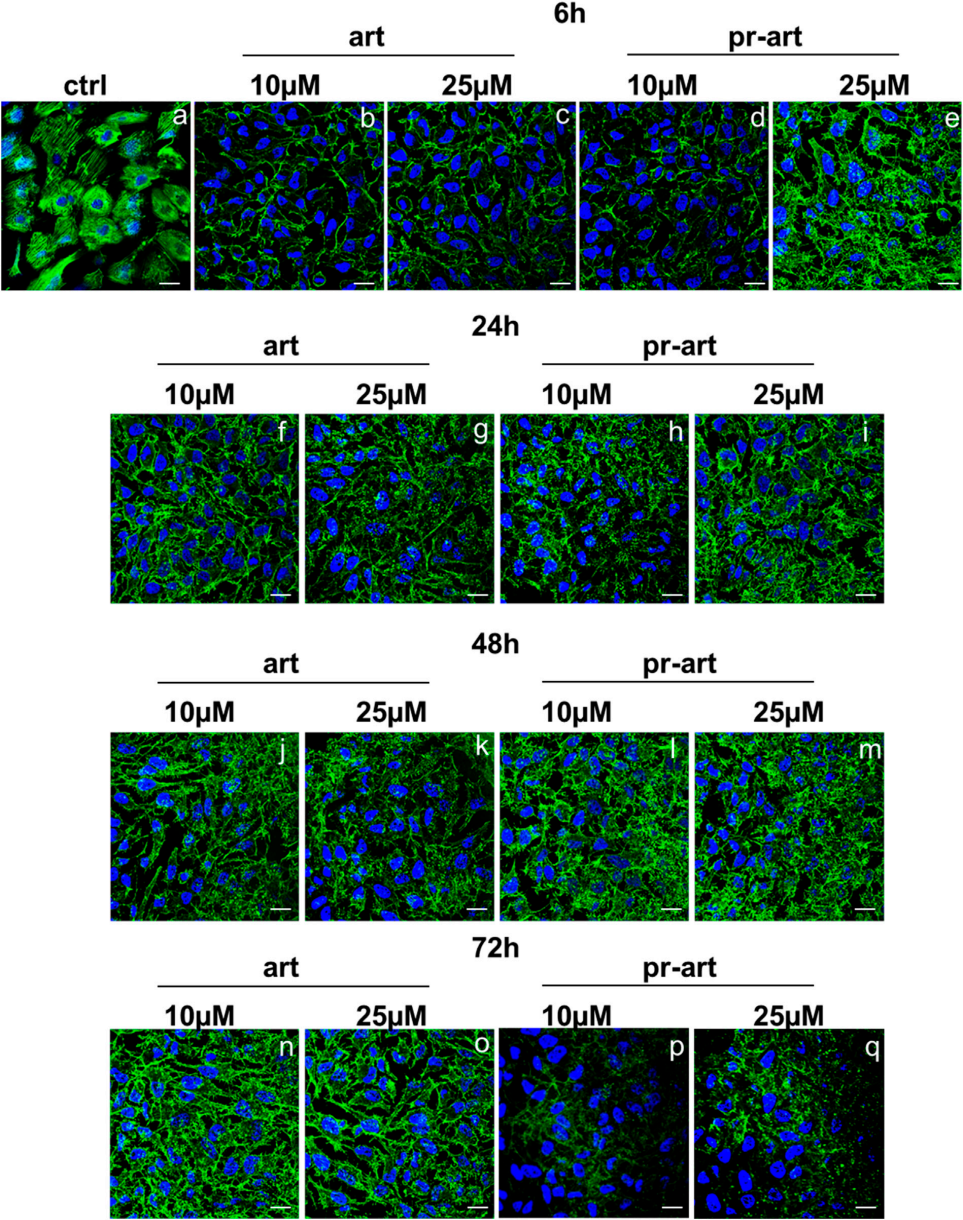
lmmunofluorescence analysis of ART and 8-p-ART-treated (10 and 25 μM; from 6 to 72 h) HeLa cells. These ones were fixed and labeled with phalloidin-FITC to detect F-actin. Magnification ×63 1.4 NA. Bar = 100 µm. All images are representative fields of n = 3 experiments with similar results.

One of the main roles of FLNA and FLNB is the connection with F-actin by which these proteins can coordinate biomechanical responses to force extracellular matrix stiffness and/or adhesive properties, with intracellular consequences (Sutherland-Smith, 2011). Here, we showed that the administration of ART and the 8-prenylated derivative to HeLa cells induced substantial disorganization of the F-actin filaments in a definitive manner compared to non-treated cells. Interestingly, these effects appeared from 6 h to 72 h of treatment. There are no differences between the two compounds and their concentrations (Figure 6, panels a–q). Actin filaments are cross-linked into bundles to form the dynamic actin cytoskeletal network, which is finely tuned by multiple families of cytoskeletal proteins belonging to the microfilament class, among which filamins represent one of the essential elements. Together with microfilaments, intermediate filaments and microtubules constitute the cytoskeleton (Yue et al., 2013). To prove the selectivity of ART and 8-p-ART on microfilaments, we performed further confocal analyses on tubulin (Supplementary Figure S3) and vimentin (Supplementary Figure S4) as representative proteins of intermediate filaments and microtubules, respectively. Taken together, these results have highlighted that the tested compounds have shown a notable selectivity for FLNA and FLNB whose disorganization could be the cause of the following disassembling of F-actin filaments. Additionally, these filaments appeared to be affected by ART and its prenylated counterpart more rapidly than filamin ones as they are characterized by a dynamic steady state conferring a high degree of plasticity to cytoskeleton networks (Plastino and Blanchoin, 2018).

### 3.6 Effects of artemetin and 8-prenylated artemetin on HeLa cell migration

**FIGURE 7:**
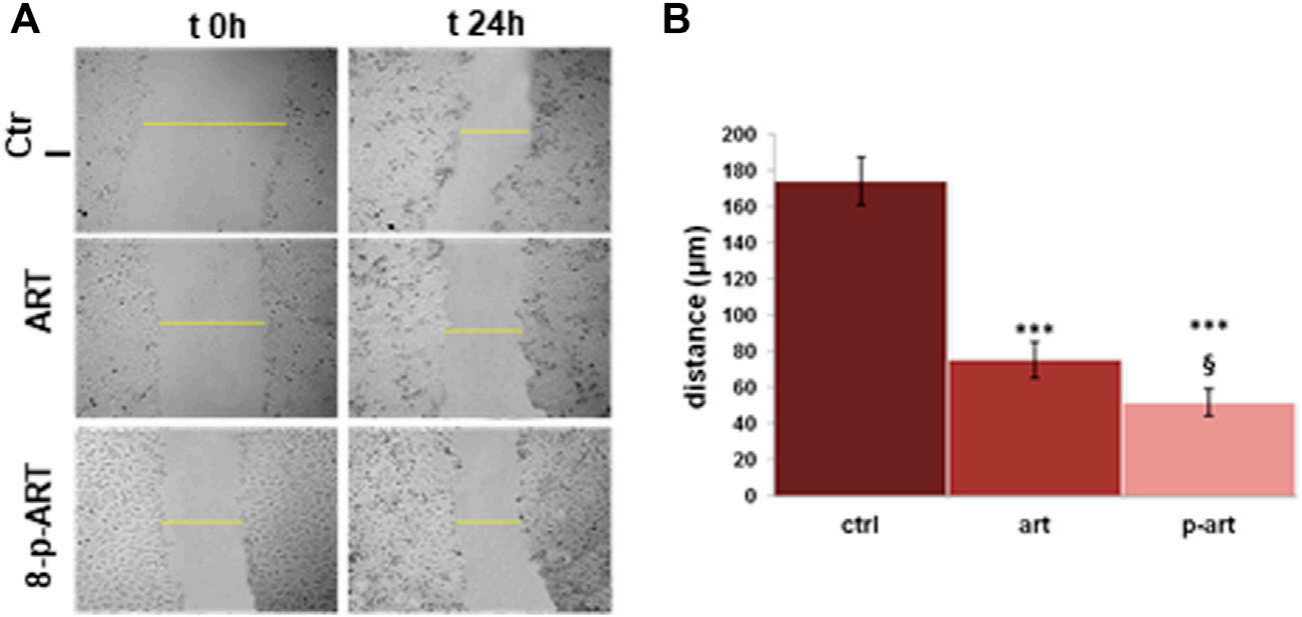
Results from the scratch wound-healing assay on HeLa cells treated or non-treated with ART and 8-p-ART at 25 µM. **(A)** Representative bright-field images of cells captured using a time-lapse microscope (Leica AF-6000 LX; Leica Microsystems). Magnification ×10. ***p < 0.001 versus untreated controls; *p* < 0.05 for 8-p-ART versus ART. **(B)** Analysis of the migration rate determined by measuring the distances covered by individual cells from the initial time to the selected time points (bar of distance tool; Leica ASF software). The data are representative of *n* = 3 independent experiments ±SD.

Cell migration is controlled by various activities combining protrusive and contractile forces, usually generated by actin filaments (Schaks et al., 2019). Since the compounds of our interest have shown a strong induction of actin disorganization, we focused on cell migratory ability, which is all well related to cancer progression (Schaks et al., 2019). By the wound-healing assay, we assessed that in the presence of ART and 8-p-ART, the migration of HeLa cells appeared notably affected. In particular, as shown by the histogram and the bright field representative images (Figures 7A,B, respectively), the prenylated compound showed a more potent inhibitory activity in replacing destroyed or damaged tissue.

## 4 Discussion and conclusion

Drug discovery development can be considered a complex picture in which bioactive compounds are settled to act on molecular targets and modulate disease-affected pathways. Therefore, drug discovery efforts look for targets or pathways that bioactive compounds can change. In this scenario, NPs are recognized as a rich source for developing anti-cancer therapies due to their unique structures and suitable pharmacological profile modulating, for instance, cell cycle and proliferation, cell survival, autophagy, and invasion pathways. Proteomic-based DARTS and LiP approaches are the golden strategies for the identification of the most reliable partners of NPs in complex mixtures of proteins from different cells or tissues, as reported in many recent publications (Morretta et al., 2021b; Ren et al., 2021). Prompted by our laboratory’s optimization of a DARTS and LiP orthogonal platform, we moved to investigate the interactome of ART in HeLa cancer cells to get a deep understanding of the anti-tumoral profile of this bioactive compound and its more permeable 8-prenylated derivative.

The anti-cancer profile of ART has been investigated since 2000 when three polymethoxyflavonoids from the fruit of *Vitex rotundifolia* were found to decrease HL-60 cell growth inducing morphological changes that are characteristics of apoptosis (Ko et al., 2000). Also, on tsFT210 cells, several flavonoids from *Vitex trifolia L.* as ART were found to inhibit the cell cycle at the G2/M phase in a dose-dependent manner with a weak induction of apoptosis (Li et al., 2005), and the anti-proliferative activity has been measured on AGS, MCF-7, and HT-29 human cancer cell lines, too (Kim et al., 2013). More recently, it has been found that ART significantly decreases tumor cell viability on three cell lines such as neuroblastoma (SH-SY5Y), hepatocarcinoma (HepG2), and nontumoral bone marrow stromal (S17) (Martins et al., 2014). For the first time, Sinha et al. (2015) showed that ART modulated microtubule depolymerization by activating mitotic spindle checkpoint working on human breast cancer cells. Molecular docking revealed that the compounds bind at the *α*-*β* interfacial site of tubulin, correlating binding interactions with inhibition. This was the first potential target disclosed for ART even though it was found by indirect evidence such as the modulation of microtubule depolymerization. Thus, encouraged by the results obtained on HeLa cells considering ART and 8-p-ART as promising anti-cancer compounds (Salamone et al., 2021; Rosa et al., 2022), we moved to identify filamins as more reliable targets using an unbiased approach.

FLNA and FLNB were disclosed as the most protected proteins by ART, and the same effect has been validated for 8-p-ART, even though filamin isoforms seem to interact with the NP by different features, both mainly in the N-terminal and C-terminal regions deputed to actin binding and their homo- and hetero-oligomerization.

Filamins are actin-binding proteins, which participate in the formation of the cytoskeleton, anchor a variety of proteins in the cytoskeleton, and regulate cell adhesion and migration (Zhou et al., 2021). Moreover, they are members of the focal adhesion protein machinery and therefore act as a linker between the ECM and the actin cytoskeleton. Indeed, by binding several partner proteins such as integrins, filamins regulate cell functions such as migration, proliferation, apoptosis, and mechanoprotection (Zhou et al., 2021). Thus, the biological effect of ART and its C8-prenylated form has been studied in detail, revealing an impact on cytoskeleton disassembly and on F-actin filament disorganization mediated by filamin dissociation, strongly induced by 8-prenylated-ART. Indeed, it is already reported in the literature that microtubule dynamics interference is an effective tool to kill tumor cells (Wordeman and Vicente, 2021): the so-called microtubule-targeting agents (MTAs) represent one of the most successful first-line therapies prescribed for cancer treatment, and they are believed to kill cells via apoptosis by blocking cell migration. Since F-actin and filamin disorders are critical elements for different fundamental cellular functions, including cell migration (Stossel et al., 2001), this phenomenon has been investigated in HeLa cells treated or non-treated with both compounds of interest, revealing that ART and 8-p-ART are potent inhibitors of cellular migration.

In conclusion, since the downregulation of cell adhesion and migration in the tumor microenvironment is a crucial step to block tumor metastasis occurrence and development, the discovery of the ART interactome by our functional proteomic platform and the insights into the interaction between the NP and its 8-prenyl derivative with filamins pave the way for consideration of this chemical scaffold as the starting point for the development of new molecules active in the treatment of cancer.

## Data Availability

The mass spectrometry proteomics data have been deposited to the ProteomeXchange Consortium *via* the PRIDE partner repository with the dataset identifier PXD034776.
