# Correction: Drug affinity-responsive target stability unveils filamins as biological targets for artemetin, an anti-cancer flavonoid

**DOI:** 10.3389/fmolb.2026.1827334

**Published:** 2026-04-29

**Authors:** Giusy Ferraro, Raffaella Belvedere, Antonello Petrella, Alessandra Tosco, Björn Stork, Stefano Salamone, Alberto Minassi, Federica Pollastro, Elva Morretta, Maria Chiara Monti

**Affiliations:** 1 Department of Pharmacy, Università di Salerno, Fisciano, Italy; 2 PhD Program in Drug Discovery and Development, Department of Pharmacy, Università di Salerno, Fisciano, Italy; 3 Institute of Molecular Medicine I, Medical Faculty and University Hospital Düsseldorf, Heinrich Heine University Düsseldorf, Düsseldorf, Germany; 4 Dipartimento di Scienze del Farmaco, Università del Piemonte Orientale, Novara, Italy; 5 PlantaChem Srls, Novara, Italy

**Keywords:** drug affinity-responsive target stability, proteomics, cytoskeleton, anti-cancer, bioactive natural compounds

There was a mistake in Figure 5F as published. An unintentional formatting error occurred in the image of 48 h treatment with Art 10 µM (panel f). The corrected Figure 5 appears below.

**FIGURE 5:**
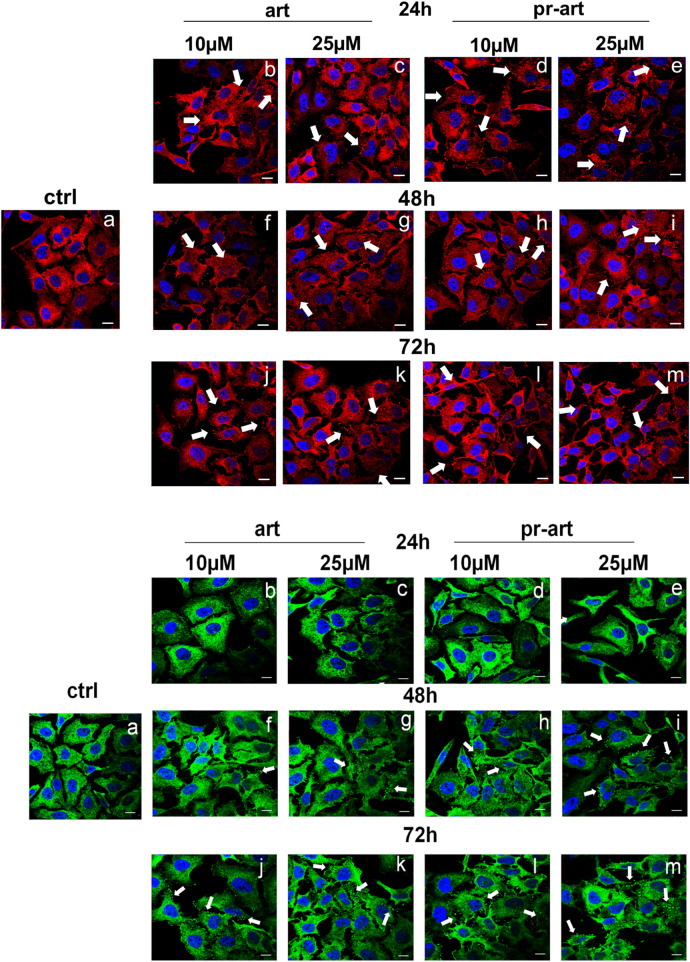
Lmmunofluorescence analysis of ART and 8-p-ART-treated (10 and 25 μM; from 24 to 72 h) HeLa cells. These ones were fixed and labeled with an antibody against FLNA (red) and FLNB (green). White arrows show FLNA and FLNB disorganization. Magnification: ×63 1.4 NA. Bar = 100 µm. All images are representative fields of n = 3 experiments with similar results.

There was a mistake in Figure 6P as published. During internal quality checks, contrast and visibility adjustments were temporarily applied using digital layers and masks to facilitate the inspection of specific features. The incomplete unintentional removal of a mask during the final export process accidentally overlapped the entire image corresponding to 72 h treatment with pr-Art-10 µM (Panel p). The corrected Figure 6 appears below.

**FIGURE 6:**
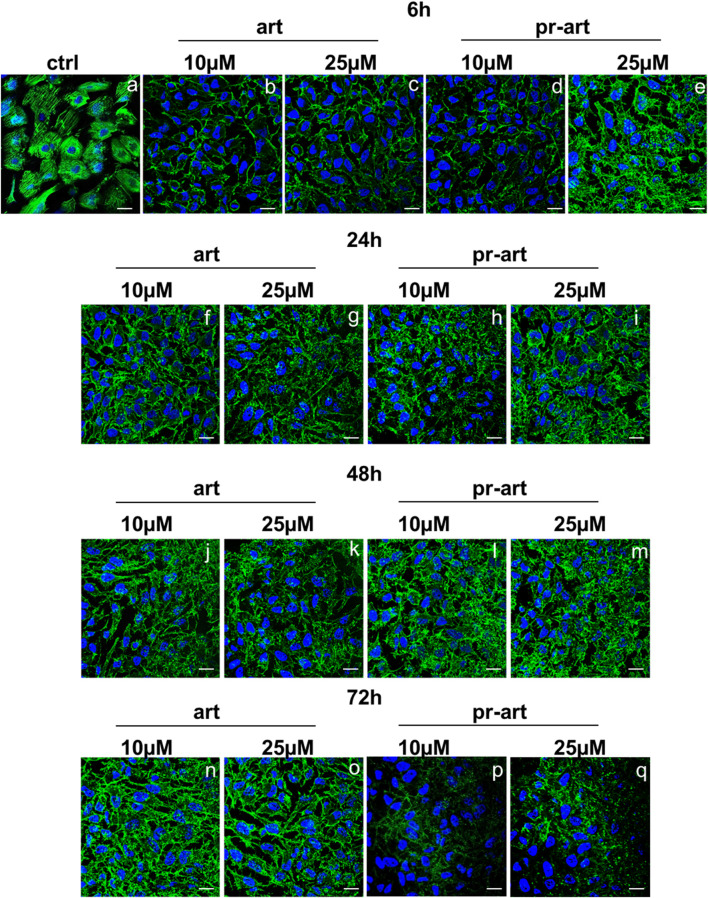
Lmmunofluorescence analysis of ART and 8-p-ART-treated (10 and 25 μM; from 6 to 72 h) HeLa cells. These ones were fixed and labeled with phalloidin-FITC to detect F-actin. Magnification ×63 1.4 NA. Bar = 100 µm. All images are representative fields of n = 3 experiments with similar results.

The original article has been updated.

