# Supplementary material for: Drug affinity-responsive target stability unveils filamins as biological targets for artemetin, an anti-cancer flavonoid
Source: Front Mol Biosci. 2022 Aug 25;9:964295. doi: 10.3389/fmolb.2022.964295 (PMC9452882; doi:10.3389/fmolb.2022.964295)
Supplement: Supplementary file 1 [file Table1.DOCX]

***Supplementary Material***

**Drug Affinity Responsive Target Stability unveils Filamins as biological targets for Artemetin, an anti-cancer flavonoid.**

# MTT assay

**
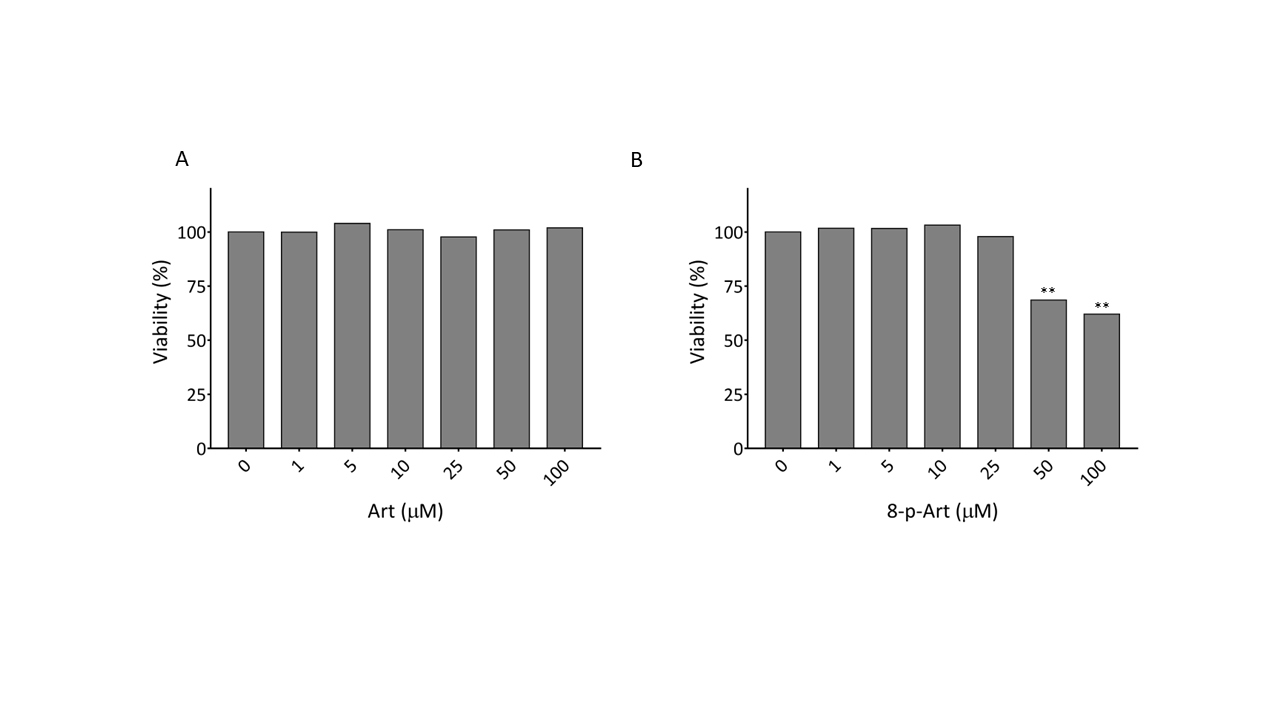
**ART and 8-p-ART cell viability was evaluated on HeLa cells through MTT assay. The experiment was performed at 24 h, 48 h and 72 h exploiting a dose-response curve from 1 µM to 100 µM of both substances. Both ART and 8-p-ART did not show any cytotoxic effect at 24h and 48h (data not shown). As reported, ART (Figure S1, A) showed no cytotoxic effects for all its concentrations, whereas 8-p-ART (Figure S1, B) negatively affected cell viability only at its highest amounts (50 and 100 µM). Thus, these concentrations were not used for the following analysis on HeLa cells.

**Figure S1** shows ART (A) and 8-p-ART (B) behavior at 72 h. Data are expressed as means of three independent experiments, setting the untreated cells viability as 100%. A Student t-test was performed (**=p< 0.01).

# Western Blots on DARTS samples

As it can be observed in Figure S2, a less remarkable proteins protection was achieved by ART on Elongation factor 2 (panel A, EF2), exportin-2 (panel B, EXP2) and DNA mismatch repair protein Msh2 (panel C, MSH2) in respect of Filamin A and B.


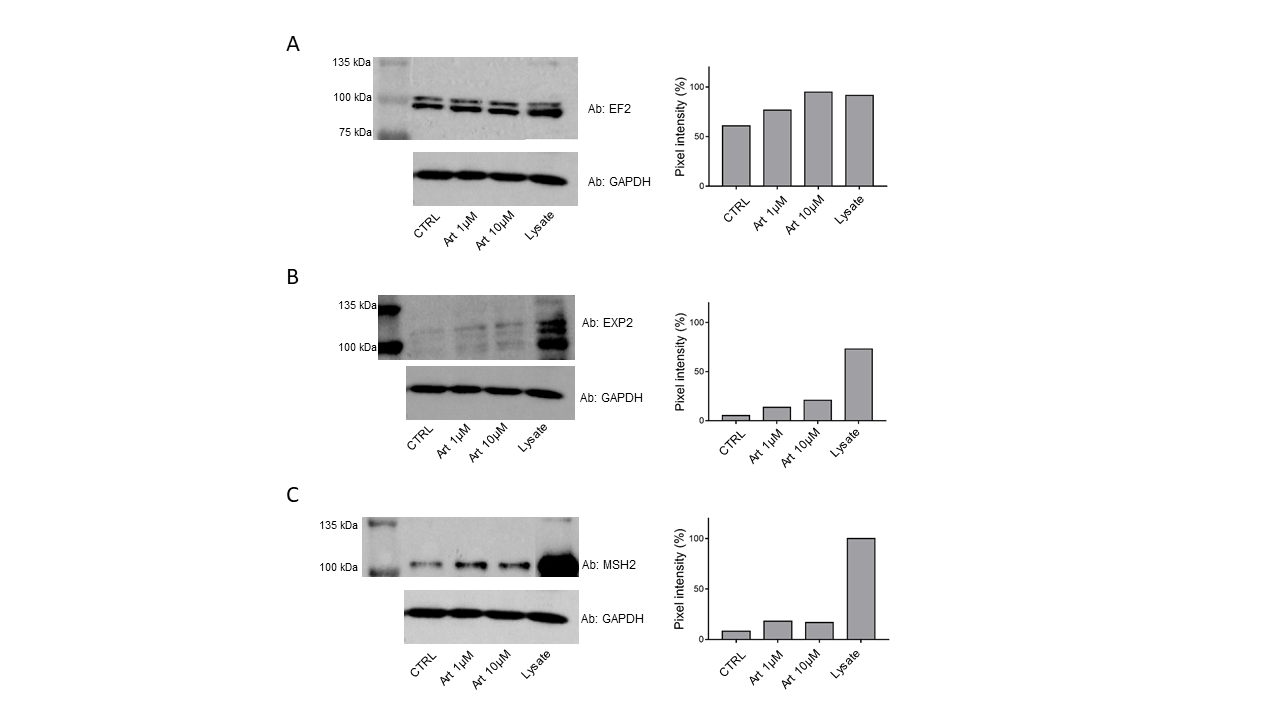


**Figure S2:** Western blotting analysis performed submitting DARTS samples to anti-Elongation factor 2 (panel A, EF2), anti-exportin-2 (panel B, EXP2) and anti-DNA mismatch repair protein Msh2 (panel C, MSH2) antibodies. GAPDH has been used as a loading normalizer to perform the densitometric analyses reported for each Blot. Here it is reported one of the DARTS experiments which were repeated twice.

# Filamin A and B Mascot results

As it can be observed in Table S1, both ART and 8-p-ART induce Filamin A and B Mascot Score, Matches and emPAI to increase in respect of the control sample.

**Table S1:** Detailed MS analysis of Filamin A and B as identified in a DARTS experiment carried out with ART and 8-p-ART, as a tile of example. The following Mascot parameters are reported: protein name (i.e., Description), Score, number of matched peptides (i.e., Matches) and exponentially modified Protein Abundance Index (i.e., emPAI). These parameters are shown for the negative control experiments (i.e., CTRL columns) followed by the corresponding ones at three ART and 8-p-ART concentrations (1 μM, 10 μM and 100 μM) and by the same values in the positive control experiments, not treated with NPs and subtilisn (i.e., Lysate columns). When a particular protein is digested by subtilisin, it is identified with low Score, Matches and emPAI in the negative control compared to the positive control, due to the proteolytic susceptibility against subtilisin. Nevertheless, when ART or 8-p-ART exposure occurs prior to subtilisin digestion, the molecule putative protein partners are identified with overall better Mascot parameters compared to the negative control, because of NPs-induced proteins stabilization and of a consequent reduction in proteolytic susceptibility against subtilisin

# t-LiP-MRM: ART protected peptides

**
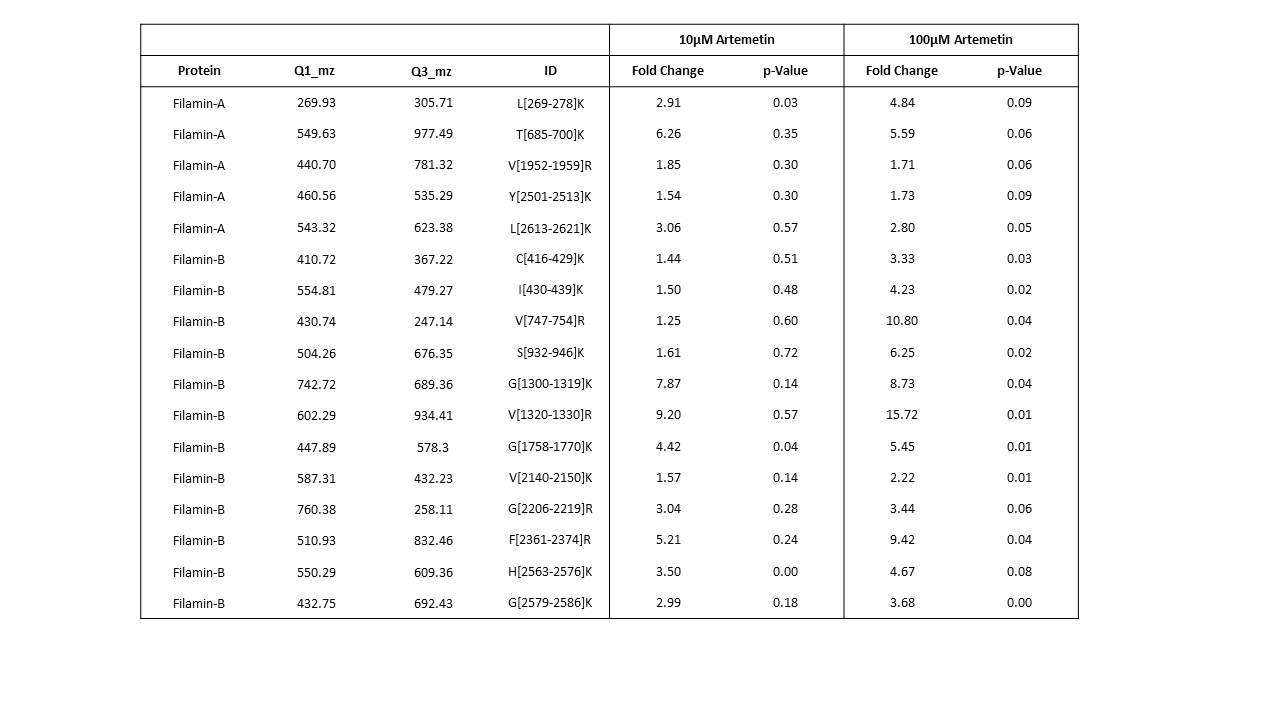
**FLNA and B tryptic peptides areas were compared between ART treated and untreated samples. Fold changes and their relative p-Values were evaluated for ART concentrations at 10 and 100 µM, in respect of the untreated sample (control). Peptides with an ART/control area fold change higher than 1.5 (p-Value < 0.1) were considered as protected from proteolysis via the interaction with ART and are reported in the table.

**Table S2:** FLNA and B regions protected by proteolysis by ART. For each peptide, the m/z of the precursor ion (Q1_mz) and of its most intense fragment (Q3_mz) are reported alongside the fold change and p-Value.

# Effects of Artemetin and 8-prenyl Artemetin on tubulin

No change in protein organization has been detected.


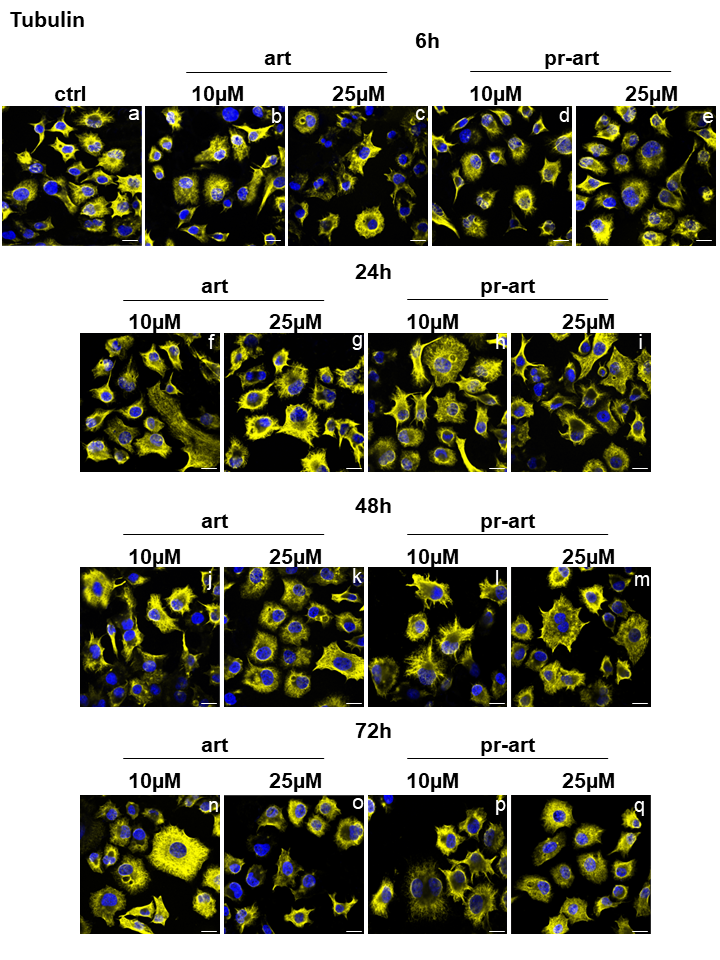


**Figure S3:** Confocal microscopy analysis of HeLa cells tubulin in presence of ART and of 8-p-ART. Treatments with both compounds were performed at 10 and 25 µM from 6 to 72 hours.

# Effects of Artemetin and 8-prenyl Artemetin on vimentin

No change in protein organization has been detected.

**
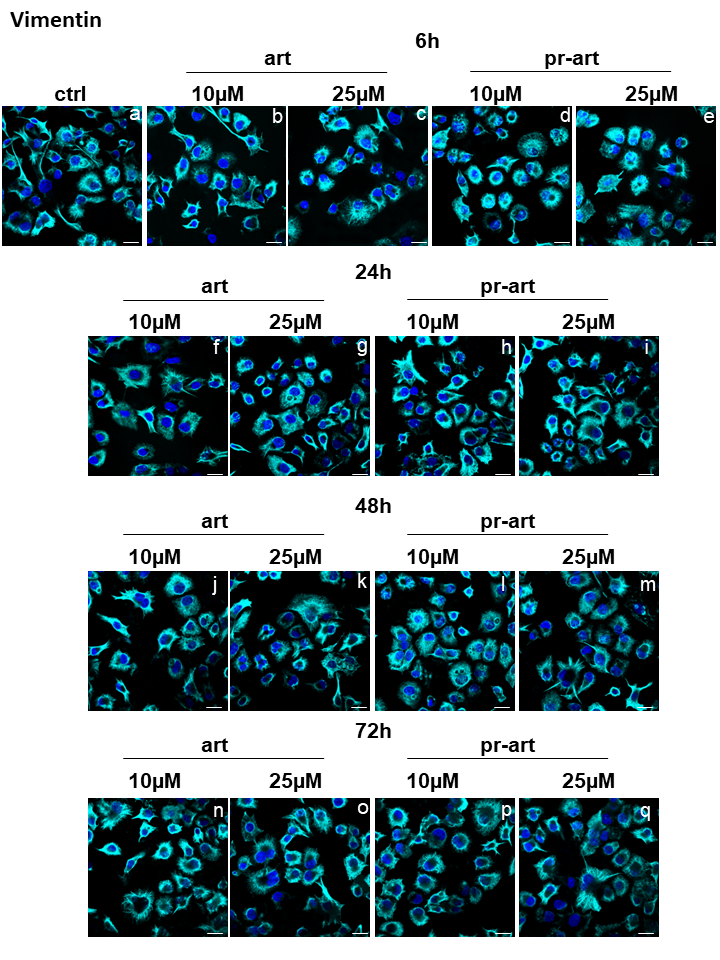
**

**Figure S4:** Confocal microscopy analysis of HeLa cells vimentin in presence of ART and of 8-p-ART. Treatments with both compounds were performed at 10 and 25 µM from 6 to 72 hours.

# Materials and Methods

**MTT assay**

ART and 8-p-ART were diluted in DMSO (from 1 up to 100 μM) and then administered to HeLa. Cell viability has been measured by MTT (3-(4,5-Dimethylthiazol-2-yl)-2,5-Diphenyltetrazolium Bromide) assay, as previously described (1). Briefly, HeLa cells were seeded at 15 × 10^3^ cells/well in a 96-well plate and incubated with ART and 8-p-ART for 24h, 48h and 72h, at 37°C. At the ends of the selected experimental times, MTT stock solution (5 mg/ml) was added to all wells of an assay (25 μl per 100 μl medium), and plates were incubated at 37°C for 3 hours. At the end of each experimental point, cells were lysed, and the dark blue crystals dissolved with 100 μl of a solution containing 50% (v/v) N, N-dimethylformamide, 20% (w/v) SDS with an adjusted pH of 4.5. The optical density (OD) of each well was measured with a microplate spectrophotometer (Titertek Multiskan MCC/340) equipped with a 620 nm filter. The viability of cells in response to treatment with tested compounds was calculated as: % viable cells = [OD (550 nm-690 nm) ART or 8-p-ART /OD (550 nm-690 nm) negative control] × 100.

**Immunoblotting analysis**

ART DARTS samples were submitted to western blotting analysis. Thus, 7 µl of each sample were loaded on an 8% SDS-PAGE and transferred onto a nitrocellulose membrane; then, they were incubated for 1h in a blocking solution (5% w/v milk in TBS-t: 31 mM Tris pH 8, 170 mM NaCl, 3.35 mM KCl, 0.05% Tween 20) and left for 16h at 4°C with monoclonal antibodies against Elongation factor 2 (EF2, RayBiotech, Inc., 1:500 v/v), exportin-2 (EXP2, RayBiotech, Inc., 1:1000 v/v) and DNA mismatch repair protein Msh2 (MSH2, RayBiotech, Inc., 1:500 v/v). Then, a rabbit peroxidase-conjugated secondary antibody (1:1000 v/v, Thermo Fisher Scientific) was added, and the signal was detected using an enhanced chemiluminescent substrate and LAS 4000 digital imaging system. Finally, an antibody against glyceraldehyde 3-phosphate dehydrogenase (GAPDH, 1:2000 v/v, Invitrogen) in 5% milk has been used as a loading normalizer.

**Confocal Microscopy**

HeLa cells were seeded on glass bottom in multiwellglass bottom plates at 8.0 x 10^4^. After treatments with either ART or 8-p-ART, cells were fixed in p-formaldehyde at 4% v/v in PBS (Lonza; Basilea, Swiss), permeabilized with Triton X-100 at 0.5% v/v in PBS (Lonza; Basilea, Swiss), blocked with goat serum at 20% v/v in PBS (Lonza; Basilea, Swiss) and then incubated with antibodies against vimentin (mouse monoclonal, 1:100 v/v; Santa Cruz Biotechnologies), and tubulin (mouse monoclonal, 1:100 v/v; Sigma-Aldrich) O/N at 4°C. The staining with conjugated secondary antibodies (1:500 v/v, anti-mouse), the nuclei with DAPI (1:1000) and the subsequent confocal microscope analysis were performed as previously described in (2,3).

# References

1. Bizzarro V, Belvedere R, Milone MR, Pucci B, Lombardi R, Bruzzese F, et al. Annexin A1 is involved in the acquisition and maintenance of a stem cell-like/aggressive phenotype in prostate cancer cells with acquired resistance to zoledronic acid. Oncotarget. 2015;6(28):25076–92.

2. Belvedere R, Saggese P, Pessolano E, Memoli D, Bizzarro V, Rizzo F, et al. miR-196a Is Able to Restore the Aggressive Phenotype of Annexin A1 Knock-Out in Pancreatic Cancer Cells by CRISPR/Cas9 Genome Editing. Int J Mol Sci [Internet]. 2018 Jul 1 [cited 2022 May 3];19(7). Available from: https://pubmed.ncbi.nlm.nih.gov/29986379/

3. Belvedere R, Bizzarro V, Parente L, Petrella F, Petrella A. Effects of Prisma® Skin dermal regeneration device containing glycosaminoglycans on human keratinocytes and fibroblasts. Cell Adh Migr [Internet]. 2018 Mar 4 [cited 2022 May 3];12(2):168–83. Available from: https://pubmed.ncbi.nlm.nih.gov/28795878/
